# Poly[[μ_2_-acetato-aquadi-μ_3_-isonicotinato-holmium(III)silver(I)] perchlorate]

**DOI:** 10.1107/S1600536809046601

**Published:** 2009-11-21

**Authors:** Sun Feng

**Affiliations:** a School of Chemistry and Environment, South China Normal University, Guangzhou 510631, People’s Republic of China

## Abstract

In the title three-dimensional heterometallic complex, {[AgHo(C_6_H_4_NO_2_)_2_(C_2_H_3_O_2_)(H_2_O)]ClO_4_}_*n*_, the Ho^III^ ion is eight-coordinated by four O atoms from four different isonicotinate ligands, three O atoms from two different acetate ligands and one O atom of a water mol­ecule. The two-coordinate Ag^I^ ion is bonded to two N atoms from two different isonicotinate anions. These metal coordination units are connected by bridging isonicotinate and acetate ligands, generating a three-dimensional network. The coordinated water mol­ecules link the carboxyl­ate group of the acetate ligand and the nitrate ligand by O—H⋯O hydrogen bonding. The crystal structure is further stabilized by hydrogen bonds. The perchlorate ion is disordered over two sites with site-occupancy factors 0.539 (12) and 0.461 (12), while the methyl group of the acetate ligand is disordered over two sites with site-occupancy factors 0.51 (4) and 0.49 (4).

## Related literature

For the applications of lanthanide–transition metal heterometallic complexes in ion exchange, magnetism, bimetallic catalysis and as luminescent probes, see: Cheng *et al.* (2006 ▶); Kuang *et al.* (2007 ▶); Peng *et al.* (2008 ▶); Zhu *et al.* (2009 ▶).
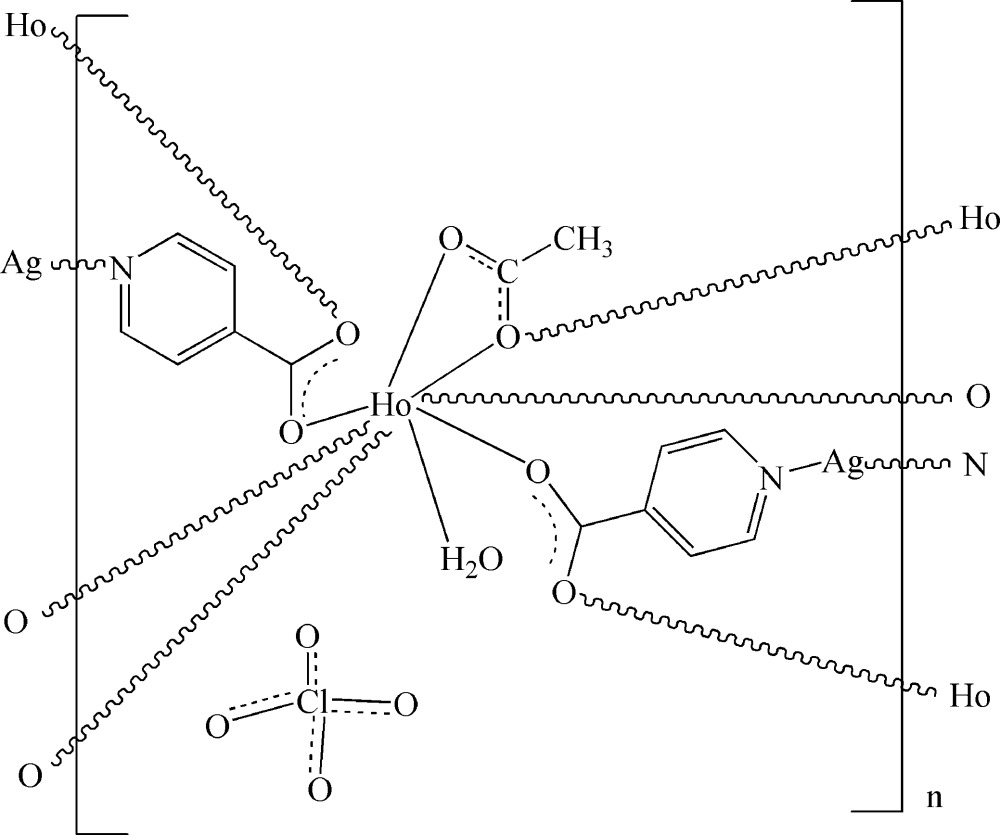


## Experimental

### 

#### Crystal data

[AgHo(C_6_H_4_NO_2_)_2_(C_2_H_3_O_2_)(H_2_O)]ClO_4_
*M* *_r_* = 693.51
Monoclinic, 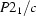

*a* = 16.2158 (10) Å
*b* = 14.9024 (9) Å
*c* = 7.9068 (5) Å
β = 91.826 (1)°
*V* = 1909.7 (2) Å^3^
*Z* = 4
Mo *K*α radiation
μ = 5.34 mm^−1^
*T* = 296 K
0.20 × 0.18 × 0.15 mm

#### Data collection

Bruker APEXII area-detector diffractometer
Absorption correction: multi-scan (*SADABS*; Sheldrick, 1996 ▶) *T*_min_ = 0.357, *T*_max_ = 0.449
9735 measured reflections
3439 independent reflections
3085 reflections with *I* > 2σ(*I*)
*R*_int_ = 0.027

#### Refinement

*R*[*F* ^2^ > 2σ(*F* ^2^)] = 0.023
*wR*(*F* ^2^) = 0.054
*S* = 1.04
3439 reflections
320 parameters
158 restraints
H atoms treated by a mixture of independent and constrained refinement
Δρ_max_ = 0.64 e Å^−3^
Δρ_min_ = −0.71 e Å^−3^

### 

Data collection: *APEX2* (Bruker, 2004 ▶); cell refinement: *SAINT* (Bruker, 2004 ▶); data reduction: *SAINT*; program(s) used to solve structure: *SHELXS97* (Sheldrick, 2008 ▶); program(s) used to refine structure: *SHELXL97* (Sheldrick, 2008 ▶); molecular graphics: *XP* in *SHELXTL* (Sheldrick, 2008 ▶); software used to prepare material for publication: *SHELXL97*.

## Supplementary Material

Crystal structure: contains datablocks I, global. DOI: 10.1107/S1600536809046601/pv2224sup1.cif

Structure factors: contains datablocks I. DOI: 10.1107/S1600536809046601/pv2224Isup2.hkl

Additional supplementary materials:  crystallographic information; 3D view; checkCIF report

## Figures and Tables

**Table 1:** Hydrogen-bond geometry (Å, °)

*D*—H⋯*A*	*D*—H	H⋯*A*	*D*⋯*A*	*D*—H⋯*A*
O1*W*—H1*W*⋯O4^i^	0.82 (4)	2.19 (3)	2.898 (4)	145 (4)
O1*W*—H2*W*⋯O6^ii^	0.81 (4)	1.99 (4)	2.787 (4)	168 (5)

Symmetry codes: (i) 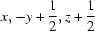; (ii) 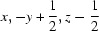.

**Fig. 1.**
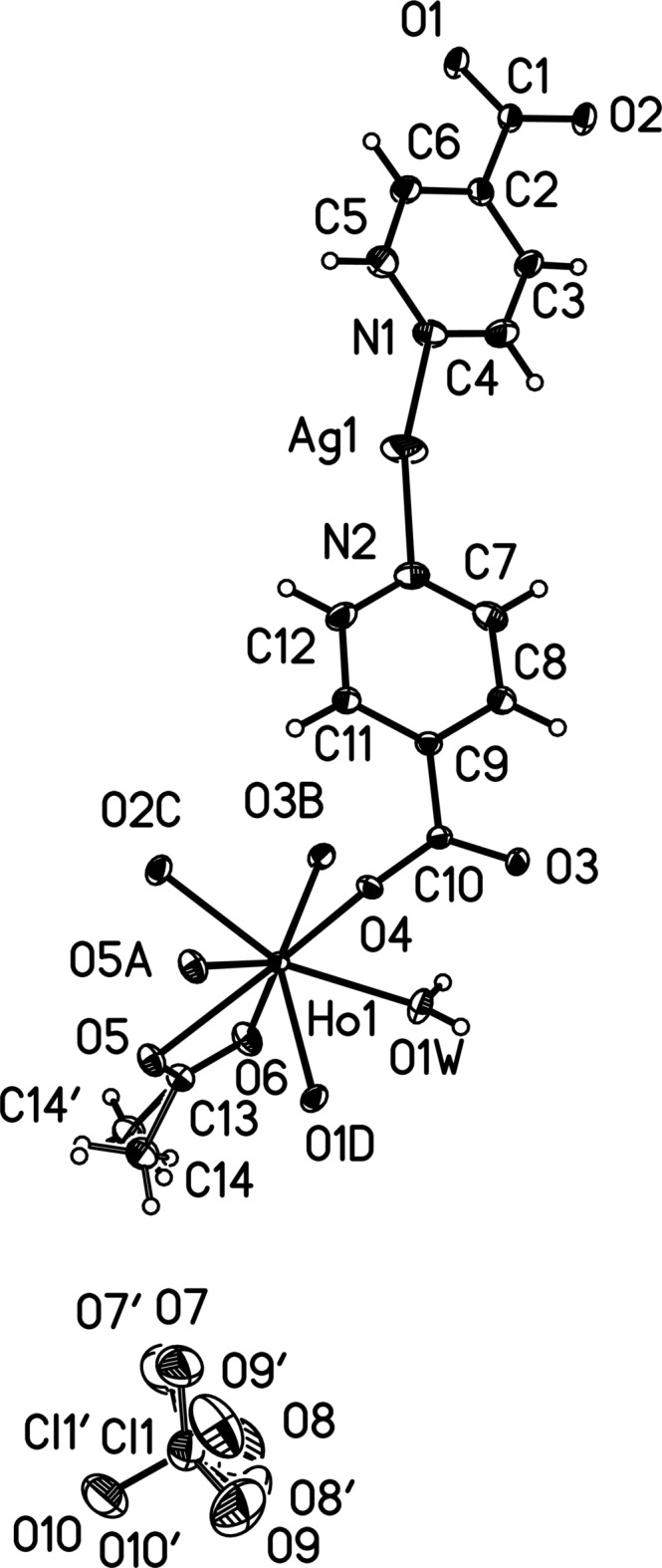
The molecular structure showing the atomic-numbering scheme and displacement ellipsoids drawn at the 30% probability level. Symmetry codes: (A) 1 - x, 1 - y, -z; (B) x, 0.5 - y, 1/2 + z; (C) -x, 1/2 + y, 0.5 - z; (D) 1 + x, 0.5 - y, -1/2 + z.

**Fig. 2.**
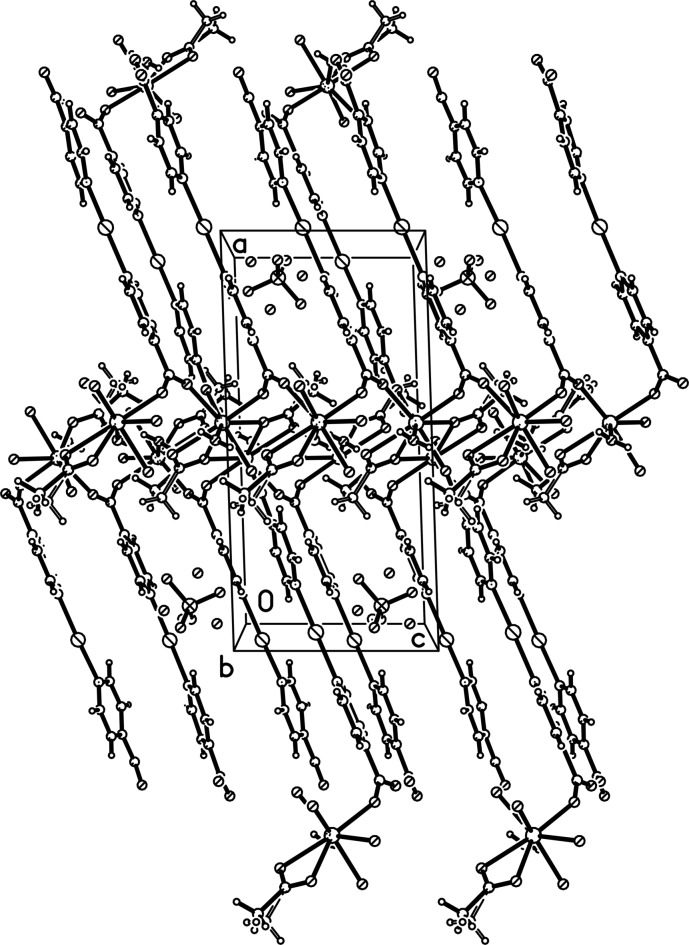
A view of the three-dimensional structure of the title compound.

## supplementary crystallographic information

### Comment

In the past few years, lanthanide-transition metal heterometallic complexs with bridging multifunctionnal organic ligands are of increasing interest, not only because of their impressive topological structures, but also due to their versatile applications in ion exchange, magnetism, bimetallic catalysis and luminescent probe(Cheng *et al.*, 2006; Kuang *et al.*, 2007; Peng *et al.*, 2008; Zhu *et al.*, 2009). As an extension of this research, the structure of the title compound, a new heterometallic coordination polymer, (I), has been determined which is presented in this artcle.

The asymmetric unite of the title compound (Fig. 1), contains one of each of the Ho^III^ and Ag^I^ ions, two halves of acetate ligand, two isonicotinate ligands, and one coordinated water molecule. The Ho^III^ ion is eight-coordinated by four O atoms from four different isonicotinate ligands, and three O atoms from two different acetate ligands, and one O atom of water molecule; the Ho center can be described as adopting a bicapped trigonal prism coordination geometry. The two-coordinate Ag^I^ ion is bonded to two N atoms from two different isonicotinate anions. Thus the Ag^I^ ion is in a somewhat liner coformation with N1—Ag1—N2 angle 165.55 (17) °. These metal coordination units are connected by bridging isonicotinate and acetate ligands, generating a three-dimensional network (Fig. 2). The coordinated water molecules link the carboxylate group and acetate ligand by O—H···O hydrogen bonding (Table 1). The crystal structure is further stabilized by hydrogen bonds.

### Experimental

A mixture of AgNO_3_(0.057 g, 0.33 mmol), Ho_2_O_3_(0.116 g, 0.33 mmol), isonicotinic acid(0.164 g, 1.33 mmol), CH_3_COONa(0.057 g, 0.7 mmol), H_2_O(7 ml), and HClO_4_(0.257 mmol)(pH 2) was sealed in a 20 ml Teflon-lined reaction vessel at 443 K for 6 days then slowly cooled to room temperature. The product was collected by filtration, washed with water and air-dried. Colorless block crystals suitable for X-ray analysis were obtained.

### Refinement

H atoms bonded to C atoms were positioned geometrically and refined as riding, with C—H = 0.93 or 0.96 Å and *U*_iso_(H) = 1.2 or 1.5 *U*_eq_(C). H atoms of water molecules were found from difference Fourier maps and refined isotropically with a restraint of O—H = 0.81 - 0.82 Å. The perchlorate ion was disordered over two sites with site occupancy factors 0.539 (12) and 0.461 (12), whereas the methyl group of the acetate ligand was disordered over two sites with site occupancy factors 0.51 (4) and 0.49 (4).

### Crystal data

**Table:**

[AgHo(C_6_H_4_NO_2_)_2_(C_2_H_3_O_2_)(H_2_O)]ClO_4_	*F*(000) = 1320
*M**_r_* = 693.51	*D*_x_ = 2.412 Mg m^−^^3^
Monoclinic, *P*2_1_/*c*	Mo *K*α radiation, λ = 0.71073 Å
Hall symbol: -P 2ybc	Cell parameters from 5514 reflections
*a* = 16.2158 (10) Å	θ = 2.5–27.8°
*b* = 14.9024 (9) Å	µ = 5.34 mm^−^^1^
*c* = 7.9068 (5) Å	*T* = 296 K
β = 91.826 (1)°	Block, colorless
*V* = 1909.7 (2) Å^3^	0.20 × 0.18 × 0.15 mm
*Z* = 4	

### Data collection

**Table:**

Bruker APEXII area-detector diffractometer	3439 independent reflections
Radiation source: fine-focus sealed tube	3085 reflections with *I* > 2σ(*I*)
graphite	*R*_int_ = 0.027
φ and ω scan	θ_max_ = 25.2°, θ_min_ = 1.9°
Absorption correction: multi-scan (*SADABS*; Sheldrick, 1996)	*h* = −18→19
*T*_min_ = 0.357, *T*_max_ = 0.449	*k* = −17→17
9735 measured reflections	*l* = −7→9

### Refinement

**Table:**

Refinement on *F*^2^	Primary atom site location: structure-invariant direct methods
Least-squares matrix: full	Secondary atom site location: difference Fourier map
*R*[*F*^2^ > 2σ(*F*^2^)] = 0.023	Hydrogen site location: inferred from neighbouring sites
*wR*(*F*^2^) = 0.054	H atoms treated by a mixture of independent and constrained refinement
*S* = 1.04	*w* = 1/[σ^2^(*F*_o_^2^) + (0.0229*P*)^2^ + 2.5125*P*] where *P* = (*F*_o_^2^ + 2*F*_c_^2^)/3
3439 reflections	(Δ/σ)_max_ = 0.001
320 parameters	Δρ_max_ = 0.64 e Å^−^^3^
158 restraints	Δρ_min_ = −0.71 e Å^−^^3^

### Special details

**Table:**

Geometry. All e.s.d.'s (except the e.s.d. in the dihedral angle between two l.s. planes) are estimated using the full covariance matrix. The cell e.s.d.'s are taken into account individually in the estimation of e.s.d.'s in distances, angles and torsion angles; correlations between e.s.d.'s in cell parameters are only used when they are defined by crystal symmetry. An approximate (isotropic) treatment of cell e.s.d.'s is used for estimating e.s.d.'s involving l.s. planes.
Refinement. Refinement of *F*^2^ against ALL reflections. The weighted *R*-factor *wR* and goodness of fit *S* are based on *F*^2^, conventional *R*-factors *R* are based on *F*, with *F* set to zero for negative *F*^2^. The threshold expression of *F*^2^ > σ(*F*^2^) is used only for calculating *R*-factors(gt) *etc*. and is not relevant to the choice of reflections for refinement. *R*-factors based on *F*^2^ are statistically about twice as large as those based on *F*, and *R*- factors based on ALL data will be even larger.

### Fractional atomic coordinates and isotropic or equivalent isotropic displacement parameters (Å^2^)

**Table:**

	*x*	*y*	*z*	*U*_iso_*/*U*_eq_	Occ. (<1)
Ho1	0.454984 (11)	0.383812 (12)	0.04996 (2)	0.01844 (7)	
Ag1	−0.02677 (3)	0.24033 (4)	0.10033 (7)	0.06287 (16)	
C1	−0.3677 (3)	0.0808 (3)	0.3622 (6)	0.0313 (10)	
C2	−0.2892 (3)	0.1236 (3)	0.3041 (6)	0.0289 (10)	
C3	−0.2191 (3)	0.0720 (3)	0.2964 (7)	0.0410 (12)	
H10	−0.2199	0.0117	0.3268	0.049*	
C4	−0.1474 (3)	0.1114 (4)	0.2427 (8)	0.0519 (15)	
H8	−0.1002	0.0762	0.2380	0.062*	
C5	−0.2111 (3)	0.2470 (3)	0.2064 (8)	0.0481 (14)	
H7	−0.2086	0.3072	0.1761	0.058*	
C6	−0.2852 (3)	0.2128 (3)	0.2588 (7)	0.0409 (12)	
H9	−0.3315	0.2493	0.2634	0.049*	
C7	0.1336 (3)	0.1719 (4)	−0.0405 (7)	0.0442 (13)	
H5	0.1066	0.1176	−0.0251	0.053*	
C8	0.2120 (3)	0.1707 (3)	−0.1043 (6)	0.0351 (11)	
H4	0.2372	0.1166	−0.1302	0.042*	
C9	0.2521 (3)	0.2510 (3)	−0.1289 (5)	0.0230 (9)	
C10	0.3375 (2)	0.2510 (3)	−0.1983 (5)	0.0206 (9)	
C11	0.2125 (3)	0.3295 (3)	−0.0872 (6)	0.0325 (11)	
H3	0.2382	0.3846	−0.1017	0.039*	
C12	0.1343 (3)	0.3254 (3)	−0.0238 (6)	0.0376 (12)	
H6	0.1080	0.3787	0.0034	0.045*	
N1	−0.1427 (2)	0.1969 (3)	0.1974 (6)	0.0458 (11)	
N2	0.0950 (2)	0.2480 (3)	0.0001 (5)	0.0394 (10)	
O1	−0.4242 (2)	0.1332 (2)	0.4072 (5)	0.0387 (9)	
O2	−0.3699 (2)	−0.0029 (2)	0.3616 (4)	0.0397 (8)	
O3	0.35906 (18)	0.18441 (19)	−0.2810 (4)	0.0303 (7)	
O4	0.38257 (17)	0.31880 (19)	−0.1691 (4)	0.0260 (7)	
O5	0.54736 (19)	0.5035 (2)	0.1583 (4)	0.0312 (7)	
O6	0.5344 (2)	0.3847 (2)	0.3129 (4)	0.0352 (8)	
O1W	0.4985 (2)	0.2318 (2)	0.0763 (4)	0.0353 (8)	
H1W	0.478 (3)	0.197 (2)	0.143 (5)	0.053*	
H2W	0.516 (3)	0.200 (3)	0.002 (4)	0.053*	
C13	0.5691 (3)	0.4589 (3)	0.2876 (5)	0.0272 (10)	
C14	0.6256 (13)	0.4991 (15)	0.423 (2)	0.040 (3)	0.49 (4)
H14A	0.5956	0.5074	0.5247	0.060*	0.49 (4)
H14B	0.6457	0.5560	0.3852	0.060*	0.49 (4)
H14C	0.6713	0.4594	0.4455	0.060*	0.49 (4)
C14'	0.6450 (11)	0.4886 (16)	0.388 (3)	0.040 (3)	0.51 (4)
H14D	0.6545	0.4486	0.4821	0.060*	0.51 (4)
H14E	0.6368	0.5484	0.4297	0.060*	0.51 (4)
H14F	0.6918	0.4877	0.3171	0.060*	0.51 (4)
Cl1	0.91931 (10)	0.45924 (11)	0.2560 (2)	0.0625 (4)	0.539 (12)
O7	0.8512 (8)	0.4643 (8)	0.3717 (18)	0.126 (5)	0.539 (12)
O8	0.8847 (9)	0.4462 (8)	0.0955 (12)	0.111 (5)	0.539 (12)
O9	0.9703 (9)	0.3896 (8)	0.300 (2)	0.130 (6)	0.539 (12)
O10	0.9589 (10)	0.5442 (8)	0.259 (2)	0.086 (6)	0.539 (12)
Cl1'	0.91931 (10)	0.45924 (11)	0.2560 (2)	0.0625 (4)	0.461 (12)
O7'	0.8366 (6)	0.4707 (8)	0.210 (2)	0.094 (5)	0.461 (12)
O8'	0.9624 (10)	0.4225 (10)	0.1137 (18)	0.132 (6)	0.461 (12)
O9'	0.9275 (11)	0.3943 (10)	0.3831 (18)	0.131 (7)	0.461 (12)
O10'	0.9581 (11)	0.5392 (9)	0.304 (2)	0.082 (6)	0.461 (12)

### Atomic displacement parameters (Å^2^)

**Table:**

	*U*^11^	*U*^22^	*U*^33^	*U*^12^	*U*^13^	*U*^23^
Ho1	0.01607 (11)	0.01592 (11)	0.02369 (12)	−0.00070 (7)	0.00617 (7)	−0.00010 (7)
Ag1	0.0259 (2)	0.0859 (4)	0.0783 (3)	−0.0142 (2)	0.0260 (2)	0.0008 (3)
C1	0.027 (2)	0.023 (2)	0.044 (3)	−0.0049 (19)	0.016 (2)	−0.004 (2)
C2	0.027 (2)	0.027 (2)	0.033 (2)	−0.0057 (19)	0.0118 (19)	−0.0029 (19)
C3	0.027 (3)	0.028 (3)	0.069 (4)	0.002 (2)	0.017 (2)	0.004 (2)
C4	0.027 (3)	0.046 (4)	0.083 (4)	0.000 (2)	0.017 (3)	−0.006 (3)
C5	0.033 (3)	0.034 (3)	0.078 (4)	−0.009 (2)	0.020 (3)	0.008 (3)
C6	0.026 (3)	0.033 (3)	0.064 (4)	−0.001 (2)	0.017 (2)	0.005 (2)
C7	0.032 (3)	0.041 (3)	0.060 (3)	−0.013 (2)	0.014 (2)	0.003 (3)
C8	0.028 (3)	0.028 (3)	0.050 (3)	−0.0040 (19)	0.012 (2)	−0.001 (2)
C9	0.019 (2)	0.027 (2)	0.023 (2)	−0.0016 (17)	0.0029 (17)	−0.0019 (17)
C10	0.018 (2)	0.022 (2)	0.023 (2)	0.0006 (16)	0.0015 (17)	0.0003 (17)
C11	0.025 (2)	0.028 (3)	0.045 (3)	−0.0027 (19)	0.012 (2)	−0.002 (2)
C12	0.028 (3)	0.035 (3)	0.051 (3)	0.005 (2)	0.015 (2)	−0.001 (2)
N1	0.025 (2)	0.046 (3)	0.067 (3)	−0.0090 (19)	0.018 (2)	−0.001 (2)
N2	0.025 (2)	0.047 (3)	0.047 (3)	−0.0028 (18)	0.0136 (18)	−0.001 (2)
O1	0.0304 (18)	0.0221 (17)	0.065 (2)	−0.0017 (13)	0.0281 (17)	−0.0023 (15)
O2	0.0346 (18)	0.0219 (17)	0.064 (2)	−0.0025 (14)	0.0279 (17)	−0.0039 (15)
O3	0.0263 (17)	0.0225 (16)	0.0431 (19)	−0.0017 (13)	0.0144 (14)	−0.0104 (14)
O4	0.0185 (15)	0.0259 (16)	0.0338 (17)	−0.0060 (12)	0.0058 (12)	−0.0054 (13)
O5	0.0382 (18)	0.0269 (17)	0.0279 (16)	−0.0076 (14)	−0.0067 (14)	0.0043 (13)
O6	0.044 (2)	0.0298 (18)	0.0315 (18)	−0.0093 (15)	−0.0022 (15)	0.0102 (14)
O1W	0.049 (2)	0.0215 (16)	0.037 (2)	0.0061 (15)	0.0189 (16)	0.0013 (14)
C13	0.028 (2)	0.029 (2)	0.025 (2)	−0.0030 (19)	−0.0014 (18)	0.0027 (19)
C14	0.039 (6)	0.044 (5)	0.036 (6)	−0.003 (5)	−0.005 (5)	−0.003 (4)
C14'	0.039 (6)	0.044 (5)	0.036 (6)	−0.003 (5)	−0.005 (5)	−0.003 (4)
Cl1	0.0599 (10)	0.0510 (9)	0.0761 (11)	0.0018 (7)	−0.0046 (8)	−0.0019 (8)
O7	0.119 (9)	0.127 (8)	0.133 (9)	−0.027 (7)	0.053 (7)	−0.013 (7)
O8	0.143 (10)	0.114 (8)	0.072 (6)	−0.050 (7)	−0.042 (6)	−0.005 (6)
O9	0.142 (9)	0.098 (7)	0.148 (10)	0.072 (7)	−0.037 (7)	−0.016 (7)
O10	0.077 (8)	0.084 (9)	0.097 (9)	−0.030 (6)	0.007 (6)	−0.010 (6)
Cl1'	0.0599 (10)	0.0510 (9)	0.0761 (11)	0.0018 (7)	−0.0046 (8)	−0.0019 (8)
O7'	0.057 (6)	0.077 (7)	0.145 (10)	0.001 (5)	−0.021 (6)	0.011 (7)
O8'	0.147 (11)	0.131 (9)	0.124 (9)	0.016 (8)	0.068 (8)	−0.035 (7)
O9'	0.143 (11)	0.133 (10)	0.117 (9)	−0.011 (8)	−0.019 (8)	0.065 (8)
O10'	0.069 (9)	0.076 (9)	0.103 (9)	−0.026 (7)	0.022 (7)	−0.045 (7)

### Geometric parameters (Å, °)

**Table:**

Ho1—O4	2.278 (3)	C9—C11	1.380 (6)
Ho1—O2^i^	2.303 (3)	C9—C10	1.504 (5)
Ho1—O1^ii^	2.306 (3)	C10—O3	1.245 (5)
Ho1—O3^iii^	2.318 (3)	C10—O4	1.264 (5)
Ho1—O5^iv^	2.352 (3)	C11—C12	1.379 (6)
Ho1—O1W	2.380 (3)	C11—H3	0.9300
Ho1—O6	2.410 (3)	C12—N2	1.335 (6)
Ho1—O5	2.465 (3)	C12—H6	0.9300
Ag1—N1	2.152 (4)	O1—Ho1^v^	2.306 (3)
Ag1—N2	2.154 (4)	O2—Ho1^vi^	2.303 (3)
C1—O2	1.248 (5)	O3—Ho1^vii^	2.318 (3)
C1—O1	1.264 (5)	O5—C13	1.260 (5)
C1—C2	1.509 (6)	O5—Ho1^iv^	2.352 (3)
C2—C3	1.376 (6)	O6—C13	1.259 (5)
C2—C6	1.379 (7)	O1W—H1W	0.82 (4)
C3—C4	1.380 (7)	O1W—H2W	0.81 (4)
C3—H10	0.9300	C13—C14'	1.510 (9)
C4—N1	1.327 (7)	C13—C14	1.511 (9)
C4—H8	0.9300	C14—H14A	0.9600
C5—N1	1.341 (7)	C14—H14B	0.9600
C5—C6	1.380 (6)	C14—H14C	0.9600
C5—H7	0.9300	C14'—H14D	0.9600
C6—H9	0.9300	C14'—H14E	0.9600
C7—N2	1.340 (7)	C14'—H14F	0.9600
C7—C8	1.382 (6)	Cl1—O9	1.364 (8)
C7—H5	0.9300	Cl1—O8	1.385 (8)
C8—C9	1.380 (6)	Cl1—O10	1.419 (9)
C8—H4	0.9300	Cl1—O7	1.458 (8)
			
O4—Ho1—O2^i^	104.08 (12)	C2—C6—H9	120.6
O4—Ho1—O1^ii^	90.36 (11)	C5—C6—H9	120.6
O2^i^—Ho1—O1^ii^	139.11 (10)	N2—C7—C8	122.7 (5)
O4—Ho1—O3^iii^	84.99 (11)	N2—C7—H5	118.7
O2^i^—Ho1—O3^iii^	74.14 (10)	C8—C7—H5	118.7
O1^ii^—Ho1—O3^iii^	146.11 (10)	C9—C8—C7	119.0 (4)
O4—Ho1—O5^iv^	76.99 (10)	C9—C8—H4	120.5
O2^i^—Ho1—O5^iv^	72.10 (11)	C7—C8—H4	120.5
O1^ii^—Ho1—O5^iv^	74.39 (11)	C11—C9—C8	118.4 (4)
O3^iii^—Ho1—O5^iv^	136.13 (11)	C11—C9—C10	121.9 (4)
O4—Ho1—O1W	78.79 (12)	C8—C9—C10	119.7 (4)
O2^i^—Ho1—O1W	148.20 (11)	O3—C10—O4	124.2 (4)
O1^ii^—Ho1—O1W	71.57 (11)	O3—C10—C9	118.0 (3)
O3^iii^—Ho1—O1W	74.60 (10)	O4—C10—C9	117.8 (3)
O5^iv^—Ho1—O1W	137.66 (11)	C12—C11—C9	119.4 (4)
O4—Ho1—O6	155.03 (10)	C12—C11—H3	120.3
O2^i^—Ho1—O6	92.45 (12)	C9—C11—H3	120.3
O1^ii^—Ho1—O6	89.10 (12)	N2—C12—C11	122.5 (4)
O3^iii^—Ho1—O6	81.64 (11)	N2—C12—H6	118.7
O5^iv^—Ho1—O6	126.62 (10)	C11—C12—H6	118.7
O1W—Ho1—O6	77.37 (12)	C4—N1—C5	117.7 (4)
O4—Ho1—O5	149.88 (10)	C4—N1—Ag1	116.4 (3)
O2^i^—Ho1—O5	74.19 (11)	C5—N1—Ag1	125.8 (4)
O1^ii^—Ho1—O5	74.55 (11)	C12—N2—C7	118.0 (4)
O3^iii^—Ho1—O5	121.89 (11)	C12—N2—Ag1	123.1 (3)
O5^iv^—Ho1—O5	73.91 (11)	C7—N2—Ag1	118.9 (3)
O1W—Ho1—O5	118.90 (12)	C1—O1—Ho1^v^	134.8 (3)
O6—Ho1—O5	52.72 (10)	C1—O2—Ho1^vi^	138.5 (3)
O4—Ho1—C13	170.02 (11)	C10—O3—Ho1^vii^	149.1 (3)
O2^i^—Ho1—C13	83.92 (13)	C10—O4—Ho1	139.5 (3)
O1^ii^—Ho1—C13	79.67 (13)	C13—O5—Ho1^iv^	160.5 (3)
O3^iii^—Ho1—C13	103.15 (12)	C13—O5—Ho1	92.9 (2)
O5^iv^—Ho1—C13	100.29 (11)	Ho1^iv^—O5—Ho1	106.09 (11)
O1W—Ho1—C13	97.63 (13)	C13—O6—Ho1	95.6 (3)
O6—Ho1—C13	26.34 (11)	Ho1—O1W—H1W	122 (3)
O5—Ho1—C13	26.45 (11)	Ho1—O1W—H2W	127 (3)
O4—Ho1—Ho1^iv^	114.60 (7)	H1W—O1W—H2W	105 (4)
O2^i^—Ho1—Ho1^iv^	68.77 (7)	O6—C13—O5	118.5 (4)
O1^ii^—Ho1—Ho1^iv^	70.43 (7)	O6—C13—C14'	122.3 (10)
O3^iii^—Ho1—Ho1^iv^	141.14 (7)	O5—C13—C14'	118.3 (10)
O5^iv^—Ho1—Ho1^iv^	37.97 (7)	O6—C13—C14	119.9 (10)
O1W—Ho1—Ho1^iv^	139.55 (8)	O5—C13—C14	120.9 (10)
O6—Ho1—Ho1^iv^	88.65 (7)	C14'—C13—C14	17.2 (11)
O5—Ho1—Ho1^iv^	35.94 (7)	O6—C13—Ho1	58.1 (2)
C13—Ho1—Ho1^iv^	62.34 (9)	O5—C13—Ho1	60.6 (2)
N1—Ag1—N2	165.55 (17)	C14'—C13—Ho1	166.4 (11)
O2—C1—O1	126.7 (4)	C14—C13—Ho1	176.2 (10)
O2—C1—C2	116.5 (4)	C13—C14—H14A	109.5
O1—C1—C2	116.8 (4)	C13—C14—H14B	109.5
C3—C2—C6	118.8 (4)	C13—C14—H14C	109.5
C3—C2—C1	118.9 (4)	C13—C14'—H14D	109.5
C6—C2—C1	122.3 (4)	C13—C14'—H14E	109.5
C2—C3—C4	118.7 (5)	H14D—C14'—H14E	109.5
C2—C3—H10	120.6	C13—C14'—H14F	109.5
C4—C3—H10	120.6	H14D—C14'—H14F	109.5
N1—C4—C3	123.3 (5)	H14E—C14'—H14F	109.5
N1—C4—H8	118.4	O9—Cl1—O8	110.5 (7)
C3—C4—H8	118.4	O9—Cl1—O10	113.8 (8)
N1—C5—C6	122.7 (5)	O8—Cl1—O10	108.2 (8)
N1—C5—H7	118.7	O9—Cl1—O7	110.2 (8)
C6—C5—H7	118.7	O8—Cl1—O7	106.8 (7)
C2—C6—C5	118.9 (5)	O10—Cl1—O7	107.0 (7)

Symmetry codes: (i) −*x*, *y*+1/2, −*z*+1/2; (ii) *x*+1, −*y*+1/2, *z*−1/2; (iii) *x*, −*y*+1/2, *z*+1/2; (iv) −*x*+1, −*y*+1, −*z*; (v) *x*−1, −*y*+1/2, *z*+1/2; (vi) −*x*, *y*−1/2, −*z*+1/2; (vii) *x*, −*y*+1/2, *z*−1/2.

### Hydrogen-bond geometry (Å, °)

**Table:**

*D*—H···*A*	*D*—H	H···*A*	*D*···*A*	*D*—H···*A*
O1W—H1W···O4^iii^	0.82 (4)	2.19 (3)	2.898 (4)	145 (4)
O1W—H2W···O6^vii^	0.81 (4)	1.99 (4)	2.787 (4)	168 (5)

Symmetry codes: (iii) *x*, −*y*+1/2, *z*+1/2; (vii) *x*, −*y*+1/2, *z*−1/2.
